# A Study on Improving Customer Value Based on the Effect of Word of Mouth

**DOI:** 10.3389/fpsyg.2021.628665

**Published:** 2021-06-11

**Authors:** Juan Fan, Lingxin Dong

**Affiliations:** ^1^School of Public Affairs, Zhejiang University, Hangzhou, China; ^2^School of Economics and Management, Beijing University of Posts and Telecommunications, Beijing, China

**Keywords:** word-of-mouth marketing, sales promotion, need matching, user adhesion, continuous customer value creation

## Abstract

The overlapping effect originates from an extension of Mendel’s law in genetics, where one of the interactions between non-alleles is called additive effect. It is more applied in studies on overlapping brand niches in marketing today, with relatively few researches on continuous customer value creation characterised by user adhesion and need matching. Based on the need matching and user adhesion that are features of the mobile Internet era, this article proposes a model for continuous customer value creation based on overlapping marketing. According to grounded theory, this article extracts three types of factors—demand effect, user effect, and overlapping marketing—that affect continuous customer value creation in the smart terminal business. From the perspective of service perception, this research explores how overlapping marketing affects product requirement matching and user adhesion based on a survey of 491 participants, and validates the theoretical model and hypotheses. It is found that overlapping marketing can effectively enhance need matching, improve user adhesion and increase customer value. This research not only addresses the confusion regarding need matching and user adhesion in the communications market, but also reveals how the smart terminal business affects continuous customer value creation in the era of the mobile Internet through overlapping marketing, combined with need matching and user adhesion.

## Introduction

With the globalisation of the economic system and market competition, the status of customers has also evolved fundamentally. Customers determine the competitiveness of a company in the market as well as its ability to continue operation. In order to have a long-term friendly relationship with customers and develop sustainable profitability, many companies pass on to customers more value that better meets their needs, thereby driving customers to repeat purchases (Rokeach, 1973; Kumar and Reinartz, 2016; Payne et al., 2017). Thus, how to use marketing tools to effectively enhance the enduring customer value when customers use products of a company has become a heated topic for marketing scholars (Wong et al., 2005). Several scholars propose sales promotion and word-of-mouth marketing, which are currently the main marketing methods (Yang and Mattila, 2016). However, they also have limitations. This article intends to use the overlapping marketing strategy of both sales promotion and word-of-mouth marketing to enhance the transfer value and perceived value of customers, and then study its influencing mechanism on the continuous improvement of customer value. Existing researches on marketing theories mostly concentrate on the enhancing effect of single methods, but rarely on the method of overlapping marketing; little research has been done on the effect of different methods on the marketing of different services at different stages. This study plans to use consumption promotion and customer adhesion as mediating variables to study the mechanism of continuous customer value creation from the joint perspective of customers and enterprises, and propose and verify a model of continuous value creation based on overlapping marketing.

## Literature Review and Research Hypotheses

### Literature Review

Current research on customer value is mainly carried out from three different perspectives. The first is to research and define customer value from the perspective of customers, mainly from the value firms create for customers (Yingzi et al., 2004). Holbrook (1996) proposed the overall assessment of the utility of a product or service after weighing the benefits perceived by the customer and the cost of acquiring the product or service and named it customer-perceived value. Kumar et al. (2000) proposed customer transfer value, that is, the difference between total customer value and total customer cost. The second is to study customer value from the perspective of enterprises, typically represented by customer lifetime value (CLV). CLV refers to the total revenue that each buyer may create for the enterprise in the future (Linjie et al., 2005). The process from the beginning of the customer–enterprise relationship to its end is a development trajectory that changes over time, which is called the customer lifecycle. It has been found that the reciprocal relationship between firms and customers, and customers’ contribution to the profits and expenses of companies exist throughout the entire customer lifecycle (Krstevski and Mancheski, 2016). The third is to study customer value from the joint perspective of customers and firms. The focus is on the value exchange process between the two, which not only realises the transactions required by customers and firms, but also forms some other economic and non-economic relationships. Few influential research results from this perspective have been achieved.

Existing research focuses on measuring the effectiveness of customer value enhancement. The definition of customer value from the perspective of customers provides sound theoretical support for customer-oriented marketing; the definition from the perspective of enterprises extends the theoretical research to generate the concept of CLV. However, the current CLV research focuses on the measurement of CLV. Jackson (1985) laid the foundation for the research on CLV measurement by first proposing a measurement model based on the traditional net present value method of measuring CLV. Many scholars have extended this research and explored the measurement of CLV (Berger and Nasr, 1998; Mihai and Salerno, 2002). Although domestic and foreign scholars have conducted extensive theoretical research and exploration regarding the measurement of CLV, it is still challenging to measure and estimate CLV in practice, mainly because of the inapplicability of current CLV measurement methods. More and more management practices have made it clear that the cost of trying to win a new customer by any means is far higher than that of maintaining an existing one. The industry is in urgent need of a method that can provide corporate practice guidance to enhance customer value.

Enterprises can make use of the sales promotion marketing method to increase the transfer value of customers. Sales promotion is a direct temptation by providing additional value or incentives to salespersons, distributors, or end users of products, with the primary goal of creating immediate sales. Kotler and Keller (2004) stated in their new version of The Principles of Marketing that sales promotion is defined as a variety of short-term incentives used to encourage purchases, promote a product, or provide a service (Kotler et al., 1996). If advertising and personal selling push customers to buy a product, then sales promotion pushes them to buy it immediately. Enterprises also use the word-of-mouth marketing method to enhance customers’ perceived value. Word-of-mouth marketing is the oldest marketing tool. As early as half a century ago, foreign scholars pointed out that word-of-mouth communication was the most important source of information for some households. Word-of-mouth marketing, supported by new technologies, is an increasingly popular research area in the international marketing community (Chae et al., 2016; Buttle and Groeger, 2017). Currently, word of mouth is also one of the most influential communication channels. Consumers generally give higher credibility to word of mouth than marketer-oriented communication (personal selling, advertising, business promotion, public relations, etc.). On the practical level of corporate marketing, word-of-mouth marketing requires that companies use a variety of effective methods to involve corporate customers in discussions about their products, services, and the overall corporate image. Firms need to encourage customers to introduce and recommend their products or services to surrounding people, thus facilitating the creation of consumption behaviour.

With the deepening of research, however, researchers have found that even though “sales promotion” and “word-of-mouth marketing” are currently the main marketing methods in terms of promoting consumption, studies in both fields have limitations (Dost et al., 2019; Yang and Mattila, 2020). The limitations of studies on sales promotion lie in the following: sales promotion only boosts short-term sales without long-term effects; frequent discounts can cause consumers to develop an inaccurate perception that the discounted price should be the reference price, so that when the price returns to normal, consumers develop averse attitudes to it; sales promotion can weaken brand reputation among consumers, destroy brand equity and encourage consumers to measure product quality by price (Brown, 2005); and sales promotion only affects the timing of purchases without affecting the purchase volume, so consumers tend to make more purchases during promotions than during off-promotion periods. The limitations of studies on word-of-mouth marketing lie in the following: theoretical research mainly emphasises the relationship and influence mechanism between word of mouth and marketing; research based on practical application focuses on how to better use word-of-mouth marketing to benefit companies; although some findings have been obtained, there is still a lack of systematic research on the control of marketing effects to explain the correlation between word-of-mouth marketing and value enhancement.

### Research Hypotheses

#### Overlapping Marketing and Need Matching

Resick et al. (2007) compared the interaction effects of personal–organizational value matching, personal–work attitude matching and personal decision-making ability matching on job satisfaction. Work attitude matching proved to have the greatest predictive effect on job satisfaction, while value matching had a certain effect and decision-making ability matching had no significant effect (Huijuan and Lirong, 2009). Kumar et al. (2000) proposed that with the emergence of product homogeneity, enterprises should carry out overall product innovation. There was an interactive relationship between core product innovation and consumers’ basic needs, and continuous innovation of formal products and additional products could meet consumers’ linear and attraction needs (Krishnan et al., 1999). Balbontin et al. (2000) argued that the link between consumer needs and product innovation required the organic integration of market-driving and market-driven models. It is critical for companies to develop both market-driving and market-driven skills, and to understand when and in what ways they should work together. Dellaert (2018) proposed that new digital technologies mean that firms need to define new marketing actions that create value for consumers who are also co-producers. Based on this, the following hypothesis is proposed:

**H1a:** Overlapping marketing promotion management has a positive impact on need matching.

#### Overlapping Marketing and User Adhesion

Yu et al. (2007) constructed a theoretical model to explain how the technical and management design attributes of virtual communities affected the perceived utility, entertainment and sense of belonging of users, thereby promoting user adhesion of virtual communities. Hallowell (1996) analysed that user satisfaction and user loyalty were the main factors affecting user profitability, while personalised services, community infrastructure, activity-based communication opportunities and perception of community circle of friends significantly affected continued user participation, and increased user adhesion with technical and management design factors (Sharda, 2009; Halverson, 2011; Tu and Yuan, 2012). Nalin (2009) proposed the social reading community as a new model of transformed traditional mobile reading. Based on an introduction to user adhesion and other concepts, the author proposed strengthening user adhesion through reviews, interaction, and viral marketing. Bansal et al. (2004) built a user adhesion model of the social reading community through five dimensions—user needs, product/service characteristics, user experience, content provider characteristics, and demographic characteristics—to aggregate user adhesion and develop a profit model supportive of its own operations. Based on this, the following hypothesis is proposed:

**H1b:** Overlapping marketing promotion management has a positive impact on user adhesion.

#### Need Matching and User Adhesion

Rowley (1996) believed that compared with traditional brand communication media, mobile terminal shopping presents new features of initiative, integration, and interaction. Starting from product catalogues, practical tools, creative games, and promotional discounts, among others, design user adhesion that meets brand demands and users’ inherent needs, focus on user experience, align content creativity with brand demands, and combine user experience with brand image to maintain and enhance the adhesion of brand users (Yeager and McGrath, 1996). Bowonder and Miyake (2000) summarised the influencing factors of industrial cluster networks into a basic platform, including the trust network, knowledge or skills network, logistics network, and environment network. The author analysed the structure of these cluster networks and studied their changes from the perspective of the general law of industrial evolution. More specifically, the process of creating network competitive edges through the connection and interaction between production networks, resource networks, and environment networks was extended to establish a better network structure, which was then associated with the deep user adhesion in the industry. Halverson (2011) posited that users were linked together through TAG, RSS, IM, or EMAIL during need matching. According to the “Six Degrees of Separation” theory, the social circle formed by user-centric connections will increasingly expand to form a strong adhesive network. Huang et al. (2015) proposed that information quality, service quality and alternative system quality had positive effects on user stickiness. Based on this, the following hypothesis is proposed:

**H2:** Need matching has a positive impact on user adhesion.

#### Need Matching and Customer Value

Shelp (1984) and Iansiti and West (1997) proposed combining technology and market in the process of emerging technology management to maximise the creation and realisation of value. In this process, many value activities were attributed to the customer value system and the customer value chain structure of emerging technologies. Bond and Houston (2003) argued that the customer value chain could eliminate the uncertainty of emerging technologies, and transform potential technologies into achievable business value, so as to meet customer needs and realise value. Huang et al. (2015) confirmed the causal relationship between the quality of products and repurchase intentions. Based on this, the following hypothesis is proposed:

**H3a:** Need matching has a positive impact on customer value.

#### User Adhesion and Customer Value

Parasuraman and Grewal (2000) argued that the context-awareness service was a kind of mobile information service, which mainly used smart terminals and intelligent networks to sense the context of users. Dai (2002) mined user data and predicted user behaviour through intelligent computing, to ultimately provide users with personalised and accurate comprehensive information services. Reichheld et al. (2000) studied the mechanism that drove customer loyalty from a lifecycle perspective, and established three main determinants of customer loyalty: customer value, customer satisfaction, and customer trust. They further revealed the relationship between the three determinants and customer loyalty as well as the customer relationship lifecycle. Morgan and Hunt (1994) revealed the dynamic characteristics of customer relationships by applying the lifecycle theory, and established the relationships between customer loyalty, customer satisfaction, customer trust, transfer costs, customer value, and customer relationship lifecycle according to the dynamic development stage. Thus, the following hypothesis is proposed:

**H3b:** User adhesion has a positive impact on customer value.

**FIGURE 1:**
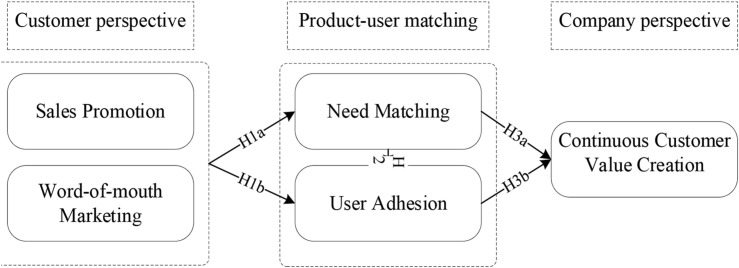
Conceptual model.

Therefore, the research model and hypotheses are proposed based on the literature review and proposed variables (see Figure 1).

## Research Method

### Measurement

#### Overlapping Marketing

This study referred to the sales promotion scale by Brown et al. (2005) and developed a theoretical marketing management scale for the traffic data user group, with a total of 8 items divided into word-of-mouth marketing and sales promotion. For the processing of the two types of data on sales promotion and ‘‘word-of-mouth marketing,’’ their product divided by 5 was used to obtain the appraisal data on overlapping marketing. The specific formula is as follows^1^:

$$O\,E\,A_{i}=\left(\frac{{\text{SPM}}_{i}}{n}\right)\times {\text{WMM}}_{i};i=1,2,3;n=5;$$

#### Need Matching

Referring to the need matching scale of Hackman and Oldham (1980), a need matching scale for traffic data user groups was developed, with a total of 5 items divided into physiological safety needs matching and social respect needs matching.

#### User Adhesion

User adhesion mainly focuses on users’ attitude and closeness to the use of traffic data, including the degree of user adhesion, such as their fondness degree, loyalty, frequency of use, customer satisfaction, and customer trust. Therefore, five questions were developed referring to the scale of agglomeration degree of Chang and Chuang (2011).

#### Customer Value

Combining the customer value scale of Parasuraman and Grewal (2000), a customer value scale for data services was developed, with a total of 4 items, including package value, traffic value, ARPU value, and voice value.

A five-point Likert scale was adopted, with 1 for completely disapprove (or totally disagree), and 5 for fully approve (or fully agree), to facilitate a comparative study of the different scales.

### Sample

The sample consisted of students using smart terminal services from universities in Sichuan, including undergraduates and postgraduates. According to the 2018 CNNIC data, 21–30-year-olds (mostly college students) account for the highest proportion of mobile Internet users in China. This article adopted the random interception method on campus to distribute surveys. To analyse the collected data, we used SPSS and WarpPLS 3.0 software. The reasons for choosing PLS were as follows: first, it could evaluate the structural model and measurement model, and measure the linear and non-linear relationships in the integrated model; second, it could measure prior variables and intermediate variables; third, it had no high requirements for the sample size and scales (Rosenthal and Rosnow, 1991; Bagozzi and Yi, 1988; Chin et al., 2003).

### Data Collection

The data collection was as follows. First, two groups were selected, with five people in each group; face-to-face interviews were conducted, mainly to see whether the items in the questionnaire could be fully understood. The questionnaire was then adjusted and modified based on the feedback to form an improved pre-survey questionnaire. Then the investigators were trained before issuing questionnaires to ensure that participants could fully and correctly understand the items in the questionnaire, and unified training on key elements was carried out. Third, a pre-survey was conducted. A total of 160 completed questionnaires were collected, of which 131 were valid, and a final questionnaire was formed based on the pre-survey results. Ultimately, a total of 561 completed questionnaires were collected for formal survey, of which 491 were valid, with an effective rate of 87.52%. Among the respondents, 57.64% were male and 42.36% were female. Most of the respondents (79.43%) were between 20 and 29 years old. 92.26% of them had 3 years of Internet use.

## Results

### Overall Index Model Analysis

In this study, the goodness-of-fit test was used to evaluate the fitness of the structural equation model (SEM) (Fornell and Larcker, 1981; Anderson et al., 1988), including the average path coefficient (APC), the average R square (ARS), and the average variance inflation factor (AVIF). The APC and ARS of the model were both significant (*p* < 0.001) and the AVIF was less than 5, indicating that the overall fitness of the model met the requirements (see Table 1).

**TABLE 1:** Model fit indices and *P*-values.

**Index**	**Model**
APC	APC = 0.275, *p* < 0.001
ARS	ARS = 0.287, *p* < 0.001
AVIF	AVIF = 1.122, good if <5

### Hypotheses Test

**FIGURE 2:**
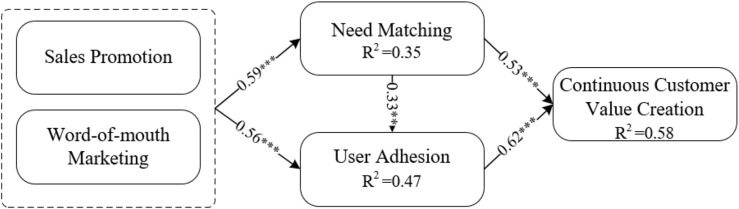
Structural equation analysis results. *p < 0.05, **p < 0.01, ***p < 0.001. *Represents the significant level.

The SEM method was applied in our data analysis. The WarpPLS 3.0 software was adopted to test and verify the hypotheses of this article. In the calculation process of the structural equation, data for the overlapping marketing appraisal were obtained from the average of the word-of-mouth marketing data multiplied by the five-point rule average of the data on the sales promotion degree. Data for the mediating variables of need matching and user adhesion and for the dependent variable of customer value were obtained from the questionnaire survey. The results show that overlapping marketing has a significant impact on need matching (β = 0.59, *p* < 0.001) and user adhesion (β = 0.56, *p* < 0.001), thus Hypotheses 1a–b are verified. Need matching has a significant impact on user adhesion (β = 0.33, *p* < 0.001), verifying Hypothesis 2. Need matching (β = 0.53, *p* < 0.001) and user adhesion (β = 0.62, *p* < 0.001) both have a significant impact on customer value, thus verifying Hypotheses 3a–b. Therefore, overlapping marketing generates significant impacts on customer value through need matching effects and user adhesion effects. The *R*^2^ value of the dependent variable represents the predictability of the theoretical model. Falk and Miller (1992) believe that the interpretation of results is valid only when the *R*^2^ value of dependent variables is greater than 10%. The model explained 35% of the variance of need matching, 47% of the variance of user adhesion, and 58% of the variance of customer value. Therefore, the research model had a good interpretability. The detailed results are shown in Figure 2.

## Discussion and Conclusion

### Conclusion

This study created a model for continuous customer value creation based on overlapping marketing, verified scales for need matching, user adhesion and overlapping marketing appraisal, and explored the impact of China’s smart terminal business on continuous customer value creation under the effect of overlapping marketing. The results proved that overlapping marketing could enhance enduring customer value creation by improving need matching and user adhesion.

### Theoretical and Practical Implications

The contributions of this study are threefold. First, this study has analysed the relationship between overlapping marketing and continuous customer value creation, building a continuous customer value creation model for overlapping marketing. Second, a method for appraising the joint effects of word-of-mouth marketing and sales promotion has been proposed, and an overlapping marketing relationship between need matching and user adhesion has been established, thereby extending theories on overlapping marketing of smart terminals.

This research also provides some practical implications. Data traffic consumers show differentiated product requirement attributes and user adhesion attributes in the information age. The communication consumers of the past mainly used fixed traditional communication services, without even trying services on emerging smart terminals. Today’s communication consumers, however, use not only a variety of different communication services, but also different data applications, hoping to satisfy a higher level of Maslow’s needs in the mobile Internet era. This pattern of product requirements and user adhesion has generated different positioning of marketing. Enterprises need to dynamically and continuously optimise product resources based on consumer needs and the characteristics of user groups to maximise the value of combining product and service resources. In addition, the ultimate source of competitive advantage lies in the overlapping and different features of word-of-mouth marketing and sales promotion in integrated marketing. Only through effectively combining these overlapping and different features, thereby implementing effective overlapping marketing, and adjusting marketing strategies, can a company maximise need matching, enhance user adhesion and increase customer value.

### Limitations and Implications for Future Research

This study has limitations due to the limited knowledge accumulation of the author and certain research conditions. First, this study adopted a dual perspective of need matching and user adhesion, but did not explore the joint effect loading matching relationship from the multiple perspectives of market environment relationships. Based on this article, subsequent research can take into account the market environment relationships to further explore the joint effect loading matching relationship. Second, some environmental factors, such as the COVID-19 pandemic, have changed consumer buying behaviour and the effect of overlapping marketing. Future research should fully take these factors into account. Third, the sample of this study consisted of college students from a single province. The sample data therefore failed to reflect the differences between groups in various regions, so the research results were not sufficiently representative. Subsequent research can include more samples and regions based on this article to further verify and deepen the conclusions of this article.

## Data Availability Statement

The raw data supporting the conclusions of this article will be made available by the authors, without undue reservation.

## Ethics Statement

The studies involving human participants were reviewed and approved by the Beijing University of Post and Telecommunications. The patients/participants provided their written informed consent to participate in this study.

## Author Contributions

Both authors listed have made a substantial, direct and intellectual contribution to the work, and approved it for publication. Both authors contributed equally to formulating the conceptual framework, analyzing the data, and writing the manuscript.

## Conflict of Interest

The authors declare that the research was conducted in the absence of any commercial or financial relationships that could be construed as a potential conflict of interest.

## Footnotes

1 OEA, overlapping effect appraisal; SPM, sales promotion management; WMM, word-of-mouth management.
